# Phenotyping of a novel *COL4A4* and novel *GLA* variant in a patient presenting with microhematuria and mildly impaired kidney function: a case report

**DOI:** 10.3389/fgene.2023.1211858

**Published:** 2023-06-01

**Authors:** Markus Ponleitner, Daniela Maria Allmer, Manfred Hecking, Constantin Gatterer, Senta Graf, Mateja Smogavec, Franco Laccone, Paulus Stefan Rommer, Gere Sunder-Plassmann

**Affiliations:** ^1^ Department of Neurology, Comprehensive Center for Clinical Neurosciences and Mental Health, Medical University of Vienna, Vienna, Austria; ^2^ Division of Nephrology and Dialysis, Department of Medicine III, Medical University of Vienna, Vienna, Austria; ^3^ Division of Cardiology, Department of Medicine II, Medical University of Vienna, Vienna, Austria; ^4^ Institute for Human Genetics, Medical University of Vienna, Vienna, Austria

**Keywords:** Alport syndrome, case report, chronic kidney disease, *COL4A4*, Fabry disease, *GLA*, microhematuria

## Abstract

We describe the case of a 44-year-old male patient with a longstanding history of microhematuria and mildly impaired kidney function (CKD G2A1). The family history disclosed three females who also had microhematuria. Genetic testing by whole exome sequencing revealed two novel variants in *COL4A4* (NM_000092.5: c.1181G>T, NP_000083.3: p.Gly394Val, heterozygous, likely pathogenic; Alport syndrome, OMIM# 141200, 203780) and *GLA* (NM_000169.3: c.460A>G, NP_000160.1: p.Ile154Val, hemizygous, variant of uncertain significance; Fabry disease, OMIM# 301500), respectively. Extensive phenotyping revealed no biochemical or clinical evidence for the presence of Fabry disease. Thus, the *GLA* c.460A>G, p.Ile154Val, is to be classified as a benign variant, whereas the *COL4A4* c.1181G>T, p.Gly394Val confirms the diagnosis of autosomal dominant Alport syndrome in this patient.

## 1 Introduction

Autosomal dominant Alport syndrome, a collagen IV disease, and Fabry disease, a lysosomal storage disease, can lead to kidney failure and other organ manifestations (Kashtan et al., 2018; Li et al., 2022). Therefore, a timely diagnosis is mandatory to allow specific treatment for the delay of disease progression.

Fabry Disease constitutes a rare X-linked lysosomal storage disorder. Pathogenic *GLA*-variants cause significant reduction in α-galactosidase A-activity in males, resulting in accumulation of the pathogenic sphingolipid metabolites globotriaosylceramide (Gb3) and globotriaosylsphingosine (Lyso-Gb3) (Germain, 2010). Disease manifestation in affected patients includes damage to the heart, kidneys and central nervous system, among others (Arends et al., 2017). Phenotype-genotype correlations allow stratification into the most severe form of Fabry disease termed “classical”-form, as well as the “late onset”-form. Depending on X-inactivation, symptoms of female patients with pathogenic variants range from no signs of disease to severe affection comparable to classic males (Echevarria et al., 2016).

Pathogenic variants in the genes encoding the different α-chains (α3, α4, α5) of collagen type IV can cause Alport syndrome, which results in a dysfunctional glomerular basement membrane with organ manifestation including microhematuria, proteinuria and progressive loss of renal function, as well as sensorineural hearing loss and ocular pathologies in case of X-linked Alport syndrome (Kashtan et al., 2018).

To the best of our knowledge the variants in the *GLA* and *COL4A4* genes detected in this patient, have not been described in the scientific literature or established databases so far. Thus, we give a detailed description of the clinical phenotype of this patient to classify his kidney disease correctly.

This description is of particular importance, because miss-classification of genetic variants would disconcert patients and may waste healthcare resources, especially in regard to expensive therapies for a rare disease.

## 2 Case description

We report on a 44-year-old male musician, born in Brazil, who had spent several years working and living in Austria at the time of diagnosis. He was referred to the Nephrology Outpatient Service at the Division of Nephrology and Dialysis, Department of Medicine III, of the Medical University of Vienna because of mildly impaired kidney function and microhematuria.

**FIGURE 1:**
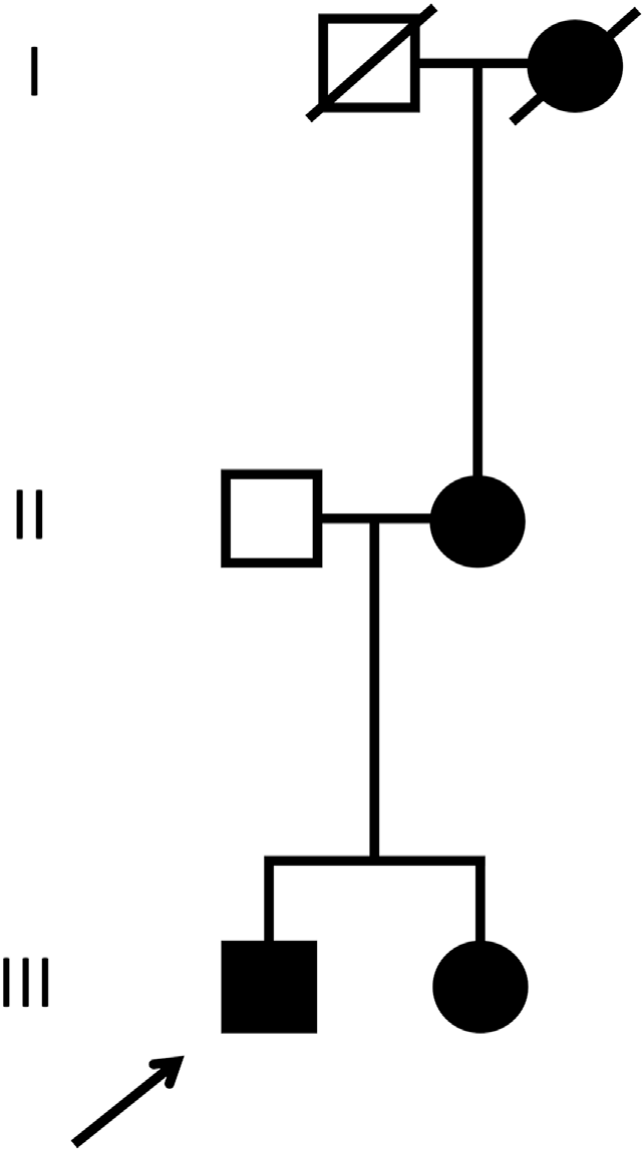
Pedigree. The patient discussed in the current case report (index patient) is marked with the arrow. Male individuals are shown as squares, females as circles. Affected (microhematuria) and healthy individuals are denoted with filled and empty symbols, respectively. Symbols of deceased individuals are crossed out.

His history disclosed the presence of microhematuria for more than 20 years. According to the patient, his sister, mother and grand-mother (mother’s side)—all born in Brazil—also suffer(ed) from microhematuria, and kidney stones. The further familial history remained unrevealing, without juvenile cardiovascular events or kidney failure (Figure 1)

During childhood, the patient had eye surgery due to strabismus. Four years before the current presentation, the patient suffered from cholecystitis and was treated with surgical cholecystectomy. Two years later, he received endoscopic retrograde cholangiopancreatography (ERCP) with stone-extraction due to gallstone-induced mild pancreatitis. No other previous illnesses were documented.

At the time we initiated the extensive workup, the patient reported no complaints and was feeling healthy. The physical examination was unremarkable, he was normotensive and has not been prescribed any medication.

## 3 Diagnostic assessment

Kidney and cardiac biomarkers over time are indicated in Table 1, confirming the presence of chronic kidney disease (CKD) stage G2A1 and persistent microhematuria, whereas serum concentrations of cardiac biomarkers were within the reference range.

**TABLE 1:** Renal and cardiac laboratory results.

Characteristic	04/12/2019	05/02/2020	19/02/2020	01/03/2021	09/05/2022	14/06/2022	19/09/2022
Age (years)	42	42	42	43	44	44	45
Serum creatinine (mg/dL)	1.14	0.97	1.09	1.11	1.25	1.09	1.11
eGFR (mL/min per 1.73m²)^a^	82	100	87	84	73	86	83
Urinary PCR (mg/g)	128	45	38	36	57	61	46
Urinary ACR (mg/g)	—	5	4	—	—	10	8
Urinary hemoglobine (mg/dL)^b^	1.0	0.5	0.5	0.5	0.5	0.5	0.1
Urinary RBC (RBC/µL)^c^	319	39	17	44	61	17	6
Troponin T (ng/L)	< 4	—	—	—	5	—	5
Pro-BNP (pg/mL)	117.3	—	—	—	13.4	—	19.5

eGFR, estimated glomerular filtration rate; PCR, protein-creatinine ratio; ACR, albumin-creatinine ratio; RBC, red blood cells; BNP, brain natriuretic peptide.

^a^ CKD-EPI 2021 equation.

^b^ dipstick.

^c^ automatic urine sediment analyzer.

Adhering to diagnostic standards in our clinic, whole exome sequencing was performed after proper patient counselling to further evaluate the suspected kidney disease in this patient and revealed two novel variants in *COL4A4* (NM_000092.5: c.1181G>T, NP_000083.3: p.Gly394Val, heterozygous, varsome.com: likely pathogenic; Alport syndrome, OMIM# 141200, 203780; ClinVar ID SCV003918893) and *GLA* (NM_000169.3: c.460A>G, NP_000160.1: p.Ile154Val, hemizygous, varsome.com: variant of uncertain significance; Fabry disease, OMIM# 301500; ClinVar ID SCV003918894), respectively (Groopman et al., 2019).

These findings pointed to a diagnosis of Alport syndrome. The variant of uncertain significance in *GLA*, however, deserved further clinical assessments. Table 2 shows details of clinical and biochemical investigation, all of which did not support the diagnosis of Fabry disease.

**TABLE 2:** Examination results.

Examination	Result	Interpretation
General		
History	Past or present Fabry pain, depression/anxiety, anhidrosis, GI-problems, dyspnea, palpitations, or chest pain were all negated	No signs of Fabry disease
PROs	No evidence of pain or reduced quality of live in respective questionnaires	Unremarkable
Height and weight	173 cm, 91 kg, BMI: 30.4 kg/m2	Obesity Class 1
Laboratory findings	TnT 5 ng/L, proBNP 19.5 pg/mL, Creatinine 1.11 mg/dL, eGFR (CKD-EPI 21) 83 mL/min per 1.73m2, CBC and coagulation studies unremarkable, LDL-cholesterol 121mg/DL, HbA1c 5.4%	Hyperlipidemia CKD G2A1, otherwise unremarkable
Ophthalmological consult	No signs of comea verticillata	Unremarkable
Dermatological consult	No signs of angioceratoma	Unremarkable
Spirometry	No sign of obstruction of restriction	Unremarkable
Hearing	No hearing loss, an audiometry was not performed	Unremarkable
Cardiac		
Blood Pressure	132/80 mmHg on-site and normal home-measurements reported by the patient	Unremarkable
Cardiac MRI	No LGE, no hypertrophy, normal LVEF, T1-Mapping, 1,000 ms	Unremarkable
Echocardiogram	Normal systolic LV-function, no value pathologies, no hypertrophy (IVS 10 mm)	Unremarkable
ECG	Sinus rhythm, 63 BPM, regular intervals and repolarization	Unremarkable
Renal		
Urinalysis	ACR and PCR within normal range, microhematuria	Microhematuria
mGFR, Cr51-EDTA	79 mL/min per 1.73m2	CKD G2A1
Renal ultrasound	Regular with a tiny spot of parenchymal calcification, no cysts	Unremarkable
Neurological		
Brain MRI	No pathological changes in gray or white matter, intracranial blood vessels of regular diameter	Unremarkable
Duplex Ultrasound of the neck vessels	Regular appearance and flow with normal intima-media thickness in carotid and vertebral arteries	Unremarkable
Fabry disease biomarkers		
α-galactosidase A activity	Leukocytes: 68 nmol/kg protein/h (normal≥51); Dried bloodspot:6.5 μmol/L/h (normal >2.8)	Unremarkable
Lyso-Gb3	Dried bloodspot: 2.1 ng/mL (normal 0-3.5)	Unremarkable

GI, gastrointestinal; PROs, patient reported outcomes; BMI, body mass index; TnT, troponin T; BNP, brain natriuretic peptide; eGFR, estimated glomerular filtration rate; CKD-EPI, chronic kidney disease epidemiology collaboration; CBC, complete blood count; LDL, low density lipoprotein; MRI, magnetic resonance imaging; LGE, late gadolinium enhancement; LV, left ventricle; LVEF, left ventricular ejection fraction; IVS, interventricular septum width; ECG, electrocardiogram; BPM, beats per minute; ACR, albumin-creatinine ratio; PCR, protein-creatinine ratio; mGFR, measured GFR; Lyso-Gb3, globotriaosylsphingosine.

## 4 Outcome

The patient received extensive counselling for his diagnosis of Alport syndrome. In addition, we provided the patient with advice regarding information for his relatives (all living abroad) and suggested they consult a nephrologist or geneticist for further consideration.

Furthermore, we scheduled doctor’s appointments annually to evaluate kidney function. To date, we did not initiate medical treatment of his disorder.

## 5 Discussion

Whole exome sequencing in this 44-year-old male patient who presented with CKD stage G2A1 and microhematuria suggested the potential presence of a kidney disease possibly caused by variants in two different genes, namely, autosomal dominant Alport syndrome and Fabry disease.

The likely pathogenic *COL4A4*-variant that was identified in the patient is located in the “triple helical domain” (consisting of repeats of the pattern Gly-X-Y) of the extra-cellular matrix protein collagen type IV-α4. The triple helical domain allows 3 type IV collagen monomers to wind together into a triple helix and form a protomer. The resulting “triple helix” mediates structural integrity and interaction with various proteins. The absence of the glycine side chain which normally lies within the helix, facing away from the surface, allows a flexible but tightly packed conformation of the triple helix. The X and Y positions in the motif are variable (often proline), are presented on the outside of the helix and can be modified. This variability in the Gly-X-Y repeat is possible without greatly affecting the stability of the triple helix. However, replacement of the glycine residue significantly affects the integrity of the triple helix and consequently the extracellular matrix, as it can no longer be packed as tightly, and protein-protein interactions and modification of the outer side chains are impaired (Fidler et al., 2018; Gibson et al., 2022).

The second genetic variant of concern in this patient, *GLA* c.460A>G, p.lle154Val, was categorized as variant of uncertain significance. The Ile154 is part of a helix of the N-terminal (β/α)_8_-barrel structure of α-galactosidase A which is located on the outside of the protein. The side chain of the affected isoleucine itself also faces more outwards and does not seem to play a role in the proteins (α-galactosidase A’s) hydrophobic core. Also, the Ile154 lies between two aspartic acid residues and itself is a hydrophobic amino acid, which is replaced by valine, another hydrophobic amino acid (Garman, 2007; Sugawara et al., 2008). Conservation analysis by MutationTaster revealed that valine also occurs at this position in *C. elegans*, whereas in *D. melanogaster* the position is occupied by lysine, a basic amino acid. According to SIFT, this position may vary in other species; in humans, chimpanzees, macaques and mice, however, the isoleucine is conserved.

Although hematuria has been described in case-reports of Fabry-patients (Solis et al., 2010; Minami et al., 2021), it is not considered a hallmark manifestation of kidney involvement due to Fabry disease, but of Alport syndrome. In line with the retained α-galactosidase A activity, no pathological findings specific for Fabry disease were obtained by extensive phenotyping of our patient (detailed in Table 2). It is therefore easily conceivable that the microhematuria in conjunction with the slightly impaired kidney function seen in our patient is not caused by Fabry disease, but instead associated with the novel *COL4A4*-variant, causing autosomal dominant Alport Syndrome (Kashtan et al., 2018). Furthermore, normal enzyme activity found in this patient permits interpretation of the *GLA*-variant as non-pathogenic (Ortiz et al., 2018).

The lack of a pathological examination of renal tissue may be considered as a limitation to our study. Indeed, a kidney biopsy would have put the results of genetic testing in better context with the renal pathology in this patient. We would have expected findings in line with Alport disease, i.e., alterations of the glomerular basement membrane and no evidence for pathological glycolipid storage, i.e., zebra bodies in podocytes and other kidney cells by electron microscopy, which occur in Fabry disease (Rumpelt, 1980; Askari et al., 2007). In light of the unimpaired α-galactosidase A-function in this hemizygous male, the kidney biopsy would not be required for establishing the diagnosis of Fabry disease. The kidney biopsy, however, was denied by the patient.

Furthermore, one could argue that the clinical work-up in a male patient with preserved α-galactosidase A activity (see Table 2) can be considered superfluous. However, given the controversial discussion on the pathogenicity of other *GLA* variants with variable enzyme activity, such as p.Arg118Cys or p.Asp313Tyr, our case should be considered informative for the medical community (Lenders et al., 2013; Ferreira et al., 2015; Oder et al., 2016; 2018; Koulousios et al., 2017; Talbot and Nicholls, 2019).

Taken together, the phenotyping of the presented case suggests classification of the novel *GLA*-variant (NM_000092.5: c.460A>G, NP_000083.3: p.Ile154Val) as benign and classification of the novel *COL4A4*-variant (NM_000169.3: c.1181G>T, NP_000160.1: p.Gly394Val) as likely pathogenic. In the context of the work-up presented in this case, the clinical phenotype of Alport syndrome associated with this novel *COL4A4*-variant seems to be mild.

## Patient consent

The patient provided written informed consent for the publication of this case report.

## Data Availability

The datasets for this article are not publicly available due to concerns regarding participant/patient anonymity. Requests to access the datasets should be directed to the corresponding author.
