# Supplementary material for: Phenotyping of a novel COL4A4 and novel GLA variant in a patient presenting with microhematuria and mildly impaired kidney function: a case report
Source: Front Genet. 2023 Jun 1;14:1211858. doi: 10.3389/fgene.2023.1211858 (PMC10267447; doi:10.3389/fgene.2023.1211858)
Supplement: Supplementary file 1 [file DataSheet1.docx]

**SUPPLEMENT**

**CARE – Checklist**

1. Title – The diagnosis or intervention of primary focus followed by the words “case report”.

– *See Title (see page 1).*

2. Key Words

– 2 to 5 key words that identify diagnoses or interventions in this case report (including "case report").

– *Alport syndrome, case report, chronic kidney disease, COL4A4, Fabry disease, GLA, microhematuria.*

3. Abstract – (structured or unstructured)

Introduction – What is unique about this case and what does it add to the scientific literature?

The patient’s main concerns and important clinical findings.

The primary diagnoses, interventions, and outcomes.

Conclusion – What are one or more “take-away” lessons from this case report?

– *Unstructured (see page 1).*

4. Introduction

– Briefly summarizes why this case is unique and may include medical literature references.

– *See pages 1 and 2.*

5. Patient Information De-identified patient specific information.

Primary concerns and symptoms of the patient.

Medical, family, and psychosocial history including relevant genetic information.

Relevant past interventions and their outcomes.

– *See page 2.*

6.Clinical Findings

– Describe significant physical examination (PE) and important clinical findings.

– *See page 2.*

7. Timeline – Historical and current information from this episode of care organized as a timeline (figure or table).

*See Table 1.*

8. Diagnostic Assessment – *See Lines 87-100.*

Diagnostic methods (PE, laboratory testing, imaging, surveys). – *See Tables 1 and 2, as well as page 2.*

Diagnostic challenges. – *NA.*

Diagnosis (including other diagnoses considered). – *see page 2.*

Prognostic characteristics when applicable. – *NA.*

9. Therapeutic Intervention

– *NA.*

10. Follow-up and Outcomes

Clinician- and patient-assessed outcomes if available. – *NA.*

Important follow-up diagnostic and other test results. – *see page 2.*

Intervention adherence and tolerability. (How was this assessed?) – *NA.*

Adverse and unanticipated events. – *NA.*

11. Discussion – *see page 3.*

Strengths and limitations in your approach to this case. – *see page 3.*

Discussion of the relevant medical literature. – *see page 3.*

The rationale for your conclusions. – *see Tables 1 and 2, see page 3.*

The primary “take-away” lessons from this case report (without references) in a one paragraph conclusion. – *see page 3.*

12. Patient Perspective – The patient should share their perspective on the treatment(s) they received.

– *NA.*

13. Informed Consent – The patient should give informed consent.

– *the patient provided prior written informed consent.*
